# Biomechanical properties of wheat grains: the implications on milling

**DOI:** 10.1098/rsif.2016.0828

**Published:** 2017-01

**Authors:** James E. Hourston, Michael Ignatz, Martin Reith, Gerhard Leubner-Metzger, Tina Steinbrecher

**Affiliations:** School of Biological Sciences, Royal Holloway University of London, Egham, Surrey, UK

**Keywords:** seed biomechanics, milling, tissue weakening, biomaterial characterization, shear force

## Abstract

Millennia of continuous innovation have driven ever increasing efficiency in the milling process. Mechanically characterizing wheat grains and discerning the structure and function of the wheat bran layers can contribute to continuing innovation. We present novel shear force and puncture force testing regimes to characterize different wheat grain cultivars. The forces endured by wheat grains during the milling process can be quantified, enabling us to measure the impact of commonly applied grain pretreatments, such as microwave heating, extended tempering, enzyme and hormone treatments on grains of different ‘hardness’. Using these methods, we demonstrate the importance of short tempering phases prior to milling and identify ways in which our methods can detect differences in the maximum force, energy and breaking behaviours of hard and soft grain types. We also demonstrate for the first time, endosperm weakening in wheat, through hormone stratification on single bran layers. The modern milling process is highly refined, meaning that small, cultivar specific, adjustments can result in large increases in downstream profits. We believe that methods such as these, which enable rapid testing of milling pretreatments and material properties can help to drive an innovation process that has been core to our industrial efforts since prehistory.

## 1. Introduction

Milling is one of the oldest ways of food processing known to mankind; the techniques and tools used for milling have been improved for centuries. Recent evidence suggests that plant starch and the grains of grasses have been milled since about 28 000 BC [1]. Milling as a technology therefore predates the spread of agriculture (the Neolithic Revolution) by more than 10 000 years. Agricultural technology has changed drastically since its beginning and modern mills are highly elaborate machines barely resembling the first known grinding stones. Wheat milling can be considered as a fractionation process and its performance is influenced by grain hardness and biochemical factors (such as protein content). The term grain hardness or endosperm texture is used to differentiate ‘soft’ and ‘hard’ hexaploid wheats and is defined as the grain's resistance to deformation. The term ‘hardness’ has a range of meanings. For material scientists, it is defined as resistance to plastic (permanent) deformation, usually measured by indentation. For engineers it can also mean resistance to wear or a measure of flow stress. To a mineralogist, it is the resistance to scratching. All of these characteristics are related to the plastic flow stress of materials. In modern bread wheat, *Triticum aestivum* subsp. *aestivum*, the hardness (or starchy endosperm texture) is among the foremost determinants of grain quality. Various measurement methods are used in food science to define the grain hardness, including single kernel characterization system, particle size index and NIR (near infrared measurement) [2–4]. Modern soft and hard wheat market classes differ genetically at the *Hardness* locus which confers the major grain texture trait decisive for the milling and flour quality [5].

**Figure 1.**
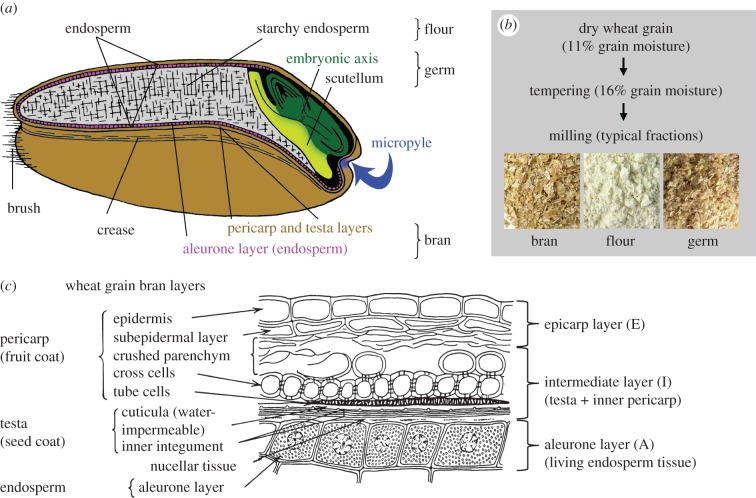
(*a*) An annotated diagram of a typical wheat grain which illustrates the parts of the seed tissue that comprise the different milling fractions (after Esau [8]). Caryopses (cereal grains) are single-seeded fruits in which the testa (seed coat) is fused with the thin pericarp (fruit coat). Cereal grains have highly developed embryos and the triploid endosperm consists of the starchy endosperm (dead storage tissue) and the aleurone layer (living cells). Organs of the cereal embryo are the coleoptile (shoot sheath), the scutellum, the radicula and the coleorrhiza (root sheath). The micropyle serves as a minute pore through which water enters. (*b*) A flow diagram illustrating the milling process from the grain's perspective. (*c*) The layers of the wheat grain separated both anatomically and functionally (after Esau [8]).

The interest in how mechanical forces can influence biological systems at different scales, in plant [6] and seed science [7], has increased within the last decade. The quality of the wheat grain milling process or more precisely the ‘scissoring’ precision for the desired bran–germ–flour separation (figure 1) depends on the distinct biomechanical properties of the wheat grain layers and their tissue-adhesive forces [9]. These properties are primarily determined by the cell-wall composition of these layers which in turn may cause distinct ‘moisture behaviour’ during the pre-milling grain tempering treatment. Tempering is used to achieve an increased grain moisture content which aids fractioning during the milling process [10]. Distinct cell-wall composition and architecture of various tissues is known in connection with their mechanical properties for different eudicot seed types and cereal grains of species including wheat [11], tobacco [12] and garden cress [13,14]. Although these seeds and grains differ considerably in structure, they share certain characteristics as they (i) have endosperm tissue adjacent or even encasing the embryo, (ii) have outer layers comprising dead tissues (testa = seed coat, pericarp = fruit coat) and (iii) have a low-hydrated state with usually only 8–12% moisture content. Cereal grains have diverse bran layers encasing the embryo and the dead starchy endosperm (figure 1) [8]. These layers comprise the living aleurone layer (which is from its ontology, part of the endosperm) and the various dead maternal bran layers which include the diverse testa and pericarp tissues (figure 1). As these layers differ in cell-wall composition and water permeability, they are the targets of pre-milling pretreatments including tempering.

The mechanical properties of wheat grains have been considered both as single grains, with multiple grains together and as individual bran layers through multiple approaches. Bending tests [15], compression [4,16–21] and tensile tests have been applied to whole grains or bran layers [22,23], respectively, often with an emphasis on moisture content [24], fracture mechanics [2,15,23] and flour quality [25]. However, the dry milling process does not allow room for large alterations in the moisture content as wheat is typically conditioned to approximately 16% moisture. To date, however, there has not been a single-grain shear testing procedure that tests grains that have been pretreated, or tempered in a way that is relevant to the milling process, nor has there been a documented puncture force testing procedure that can test individual bran layers.

Flour quality and milling yield are known to be influenced by the moisture content of the wheat grains [25]. *In vivo* ^1^H-NMR microimaging (MRI) during cereal grain imbibition suggests several preferred pathways for water uptake which include the micropyle as an opening, the embryo and scutellum as water-distribution organs, and parts of the bran layers which allow fast water uptake during the very early phases of wheat imbibition [26]. This non-uniform uptake and distribution of water has also been observed in other types of seed [27]. Tempering is a standard procedure today to increase and adjust the moisture content of wheat, often to around 16% before milling. Adjustment of moisture content is usually done by spraying water onto the wheat grains to modify the mechanical properties of grain tissues. This process of starchy endosperm mellowing prior to milling is very important for the production of high-quality flour. However, there is still not much known about the processes within the grain, which take place during the tempering period with water. Tempering serves different purposes. It is used to reduce the power consumption of mills by softening (mellowing) the starchy endosperm and toughening the bran skin, thus preventing it from being broken into small particles which would lead to higher mineral content and darker flour. Moisture content as well as temperature influences bran fractioning as the extensibility of the bran layers is altered or the brittleness increased [23,25]. The distinct cell-wall composition of the wheat grain tissues together with a cuticle as water uptake barrier (figure 1*b*) contribute to a non-uniform moisture distribution even after the tempering process is completed [10]. Additionally, MRI has revealed in high-glucan containing cereal grains that the cell-wall polysaccharide composition of grain tissues alters the water uptake pathways [28]. It is generally agreed that tempering softens the starchy endosperm and toughens the bran layers which together aid the milling process [10], but mechanistic insight into the mechanical details of this process and its relatedness to altering the biophysical properties of the various grain tissues is scarce. The addition of auxiliaries (e.g. enzymes or plant hormones) to this water is one promising way to further improve the milling quality and yield.

The differences between soft (for cookies) and hard (for bread) wheat in starchy endosperm texture (grain hardness) [5,10] have profound implications for the ideal tempering time and conditions to permit the best milling. Infrared hyperspectral imaging has been used to monitor the diffusion of water during tempering into single wheat grains differing in hardness [29]. These findings showed that water uptake into soft grain starchy endosperms is faster than into hard grain types. This is caused by enhanced capillary forces due to lower adhesion between the starch granules and the protein matrix of soft grain starchy endosperms. In wheat cultivars, this texture trait is determined by the *Puroindoline a* (*Pina*) and *Puroindoline b* (*Pinb*) gene alleles at the *Hardness* locus (*Ha*) [5]. Although the genetic determinants that distinguish soft and hard wheat are known [30,31], what is known in less detail is the relationship between the genes and the biochemical and biomechanical basis for the grain hardness differences. Soft and hard wheat starchy endosperms differ in their puroindoline a (PINA) and b (PINB) protein contents, but how this translates into defined biomechanical differences that underpin the distinct milling and flour properties is still unclear. This is, at least in part, due to the lack of appropriate biomechanical technologies to analyse single grain and individual tissue forces. Understanding the process of starchy endosperm mellowing of wheat grains during tempering and its underlying biomechanical principles will help to improve milling efficiency (e.g. shorten the tempering time, saving energy). Having a toolkit available to study the influence of physical, mechanical, chemical or enzymatic characteristics of wheat grains and bran layers will contribute to this understanding.

The aim of this study was to create a suite of novel single-grain techniques to allow for the biomechanical characterization of wheat starchy endosperm mellowing and of bran layer changes. To develop these methods, two winter wheat cultivars were selected, Cambrena (a soft wheat) and Runal (a hard wheat). With this aim in mind, our objectives were twofold (i) to assess using shear testing whether different tempering times and pre-milling treatments affect the maximum force, energy and breaking behaviours of the starchy endosperm and (ii) to investigate, using puncture force, the effect of different moisture contents and hormone and enzyme treatments on the individual bran layers of wheat grains.

## 2. Material and methods

The cultivars selected for optimizing biomechanical characterization techniques were the ‘soft’ biscuit wheat, Cambrena, with samples taken from two provenances (1725: Posieux FR, 8212: Neuhausen SH, both in Switzerland), alongside Runal, a ‘hard’ inland top wheat from three provenances (2852: Courtételle JU; 3052: Zollikofen BE; 4533: Riedholz SO, all three in Switzerland). Characteristic properties of the wheat grains are summarized in table 1. Seed weight was determined by the 1000 kernel weight and dry moisture content by a moisture analyser (*n* = 20, 2.5 g subsample each, HB43-S, Mettler-Toledo Ltd, UK). The vitreousness of the starchy endosperm was determined by optical characterization of a transverse sectional area of the grains using a microscope (Leica MZ 125, Leica Microsystems, UK). Grains were classified as glassy/mealy if more than 75% of the starchy endosperm showed the corresponding characteristic. Grains exhibiting both glassy and mealy characteristics have been classified as mixed (*n* = 300 grains per provenance). Total protein contents have been determined via NIR reflectance spectroscopy (ICC Standard Method No. 159) and soluble protein content was determined by a Bradford assay (*n* = 5 with 25 starchy endosperm samples for each provenance [32]. To prepare samples for the Bradford assay, ground starchy endosperm samples (100 mg) were freeze dried, homogenized (Precellys24, Bertin Corp., USA), extraction buffer added (pH 18, 100 mM TRIS, 10% glycerol, 0.25% SDS, 0.1% Tween20, 2 mM DTT, 150 mM NaCl), vortexed, heated (5 min, 95°C), cooled (3 min, ice) and centrifuged (15 min, 14 000 r.p.m., 4°C) before the supernatants were transferred to be used in the assay.

**Table 1.** Grain characteristics of Cambrena and Runal wheat. Data have been pooled for the different provenances as there was no difference found across the same cultivar.

	1000 seed weight (g)	moisture content of dry grain (%)	vitreousness (%)			total protein (%)	soluble protein (%)
			mealy	mixed	glassy		
Cambrena	41.01 ± 2.6	12.5 ± 0.03	76.2	17.5	6.3	10	1.29 ± 0.02
Runal	46.07 ± 0.57	10.2 ± 0.09	3.9	14	82.1	15.5	1.38 ± 0.02

### 2.1. Wheat grain tempering treatment

Prior to carrying out biomechanical tests on wheat grains a tempering phase was conducted to accurately mimic the industry standard process of mellowing prior to milling. A 2.5 g subsample of wheat grains was processed in a manual burr grinder for 2 min. The processed subsample was then transferred to a moisture analyser (HB43-S, Mettler-Toledo Ltd, UK) and its moisture content was measured. The moisture content of the remaining subsamples was adjusted to 16% (w/w) via the addition of further deionized water. Twenty grams of wheat grains were shaken vigorously for 3 min at 20°C with the calculated addition of water in a sealed Petri dish. Subsequently grains were incubated for a specified tempering time, as indicated.

Cambrena 1725 and 8212 grains were measured after 0, 1 and 6 h tempering times. Runal 2852, 3052 and 4533 gain samples were measured after 0, 1, 6, 12, 18 and 24 h to represent typical tempering times for these cultivars (*n* ≥ 26).

Runal 4533 grains were selected for further testing to explore the effects of various pretreatments on the shear force required to shear the starchy endosperm. After 6 h tempering Runal 4533 grains were divided between eight groups and subjected to a series of different conditions (*n* = 12):

(1) Control (tempering at 20°C with distilled water to adjust 16% grain moisture).
(2) Heat (35°C).
(3) 10 µM GA (gibberellin A_4+7_, Duchefa, in 0.005% (w/v) DMSO).
(4) Microwave (20 g wheat material was microwaved 1 h after the tempering at low power (520 W) for 20 s (open lid). Temperature was checked during the experiment and recorded at 54°C).
(5) Pressure (1 h, 30 bar for 30 min).
(6) Vibration (Vortex-Genie^®^ 2, shaking motion: orbital, orbit: 4 mm, amplitude: 2 mm, max. speed: 2700 rpm, for 32 s).
(7) Vacuum (vacuum desiccator VWR 467-0102 DIN, negative pressure: −72 ± 6.8 MPa).
(8) Vacuum followed by the tempering step (vacuum desiccator VWR 467-0102 DIN, negative pressure: −70.1 ± 10 MPa).

### 2.2. Shear testing

A shear testing regime was developed to mirror the type of forces that wheat grains undergo during the milling process. This method determines the force and energy that is required to shear the starchy endosperm (until break). This novel procedure allows for very precise measurements of some of the key forces involved in the milling processes.

**Figure 2.**
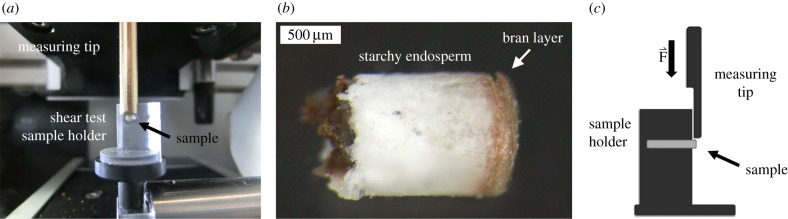
Shear force testing of wheat grain samples. (*a*) Custom built sample holder for shear tests. The sample is placed in a 1.2 mm hole in a shear test sample holder while a measuring tip is lowered and shears the sample. (*b*) Prepared cylindrical wheat grain sample with 1 mm diameter. (*c*) Schematic of the shear testing set-up.

The shear tests were conducted with a custom-made testing machine with a load cell range 0–20 N. This machine has the advantage of a modular set-up the force sensor, sample holders and optical analyses can be re-customized as appropriate. Samples can be positioned via an *x*–*y* stage (LTM80, OWIS^®^ GmbH, Germany), accuracy of 5 µm. The needle was driven on the *z*-axis which was powered by a HVM60 DC servo-motor with an accuracy of 6.75 µm (OWIS^®^ GmbH, Germany). Additionally, a high-resolution camera (1024 × 768, Stingray Allied Vision Technologies, Germany) is attached to the machine to optically track deformation. The application of custom built sample holders for shear tests was implemented (figure 2). Cylindrical samples 1 mm in diameter were punched out of the tempered wheat grain endosperm (hollow punch, Ø 1.0 mm, www.locheisen.com). These cylinders were adjusted to be 1.3 mm in length using a razor blade (as measured from the bran towards the starchy endosperm) and had a 1 mm diameter. These cylinders were then checked for pre-existing fractures under a dissection microscope (Leica MZ 125, Leica Microsystems, UK). Samples with pre-existing fractures were discarded but these samples constituted approximately less than 10% cylinders. The cylinders were placed in custom-made specimen holders (1.2 mm hole in the Plexiglas sample holder) and sheared. The measuring tip was lowered at a speed of 1.4 mm min^−1^ to the sample at a constant speed, while force and displacement were recorded. All testing was conducted at a constant 20°C.

The application of this shear testing method allows for the calculation of several biomechanical indices. Firstly, the max force recorded prior to the breaking point of the endosperm was measured and secondly, the shear strength of the material was calculated, as the cross-sectional area of the punched specimen is constant and known (*π*/4 mm^2^). The physical work that was done in separation of the respective layers and the energy needed was calculated by integrating the area enclosed by the force as the energy dissipation. Finally, the force displacement curves were interpreted and classified as to the breaking behaviour (failure type). We split this biological material into three discrete categories of breaking behaviour (i) a ‘brittle’ failure: force increases until a sudden complete failure takes place, (ii) a ‘composite’ failure: a step-by-step failure after reaching the peak force, creating a typical ‘zig-zag’ pattern and finally, (iii) a ‘continuous’ failure: typified by an increase in force followed by a continuous decrease in force without sudden drops or steps. Example curves for all three types of breaking behaviour can be found in figure 4*c*. Using this shearing method, it was also possible to shear off the bran individual layers from the cylinder samples. We present some characteristic sample curves to demonstrate the feasibility of this approach.

### 2.3. Puncture force testing

To test the force required to puncture the individual wheat grain layers, the layers of Runal 4533 were separated using the methods laid out by Antoine *et al.* [22]. To prepare the samples the brush and germ of the wheat grain was cut away and the remaining section of the grain was soaked in deionized water for 12 h. After soaking, bran layers were manually cleaned from starch and then the bran was separated into three experimental layers by sliding a preparation needle between them [22]. The isolated layers were the aleurone (A), the intermediate layer (I) and the epicarp (E) (figures 1,5,6, *n* ≥ 11).

The separated layers were flattened and left to dry on microscope slides under coverslips for 10 h. Samples were assigned to different treatment groups, an untreated control, two hormone treatments and an enzyme mixture. Following the addition of these treatments, samples were incubated for 22 h at 32°C in darkness. Controls were incubated in deionized water (1 ml). The two hormone treatments consisted of a treatment of gibberellin A_4+7_ (GA) and one of *cis*-S(+)-abscisic acid (ABA, Duchefa) which were used at final concentrations of 10 µM. The enzyme cocktail contained equal volumes of cellulase—Sigma-C2605 (1000 U ml^−1^); pectinase—Sigma-P2736; pectinesterase—Sigma-P0764; Viscozyme L—Sigma-V2010; xylanase—Sigma-X2753 (2500 U g^−1^) used at concentrations of 1%, 0.1% and 0.01% (v/v), respectively. The water content of all samples was equilibrated above a saturated NaCl/KCl mixture at 20°C.

A second experiment was conducted on the single layers in order to assess the influence of water content on the force required to puncture the separated layers. Two moisture content treatments were then created, one with a higher water content (approx. 16% grain moisture) and one at a lower water content (approx. 5% grain moisture). This was achieved by placing samples above a saturated salt solution for 10 h. A high moisture content was created using a saturated NaCl/KCl mixture at 20°C and a low moisture content was created using saturated LiCl solution at 10°C.

**Figure 3.**
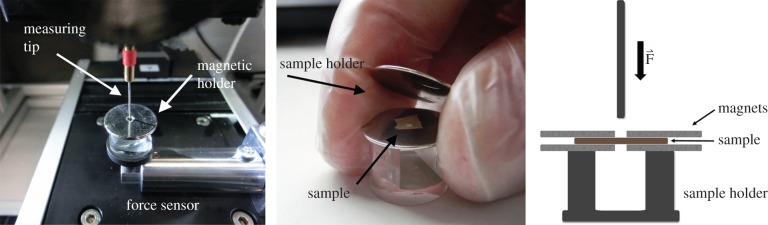
Custom-made sample holder for wheat bran layers. Separated layers are kept in place by two magnets. (Neodymium magnet, vertical pull 0.1 kg.) Measuring tip: rounded pin with 0.5 mm diameter.

The actual puncture force measurements of the samples were conducted with a custom-made testing machine with a load cell range 0–1 N. The layer was placed in a custom-made magnetic sample holder and a 0.5 mm diameter probe (hemisphere shaped tip) was lowered at a speed of 0.7 mm min^−1^ onto the layer (figure 3) while force and displacement were recorded simultaneously.

### 2.4. Data analysis

All analysis was carried out using R v. 3.2.3 ‘Wooden Christmas-Tree’ [33]. ANOVAs were used to analyse the data collected in these experiments and all post hoc contrasts were carried out using Tukey's honest significant difference test. Models were simplified through Akaike information criterion (AIC) testing and only results from minimal models are reported.

## 3. Results

### 3.1. Grain characteristics

The two wheat cultivars studied differed in their weight, dry moisture content, vitreousness and protein content, however, there was no difference found across different provenances of the same cultivar (table 1). Runal, the hard wheat, had heavier grains, a lower dry grain moisture content, a higher percentage of glassy starchy endosperms and a higher protein content, when compared with Cambrena, the soft wheat.

### 3.2. Comparative shear force testing of soft and hard wheat grain tempering

To quantify the shear forces involved in the milling process, a series of novel protocols were developed to provide a robust and repeatable testing environment. This was achieved using a number of customized components and machines engineered in-house (figure 2).

**Figure 4.**
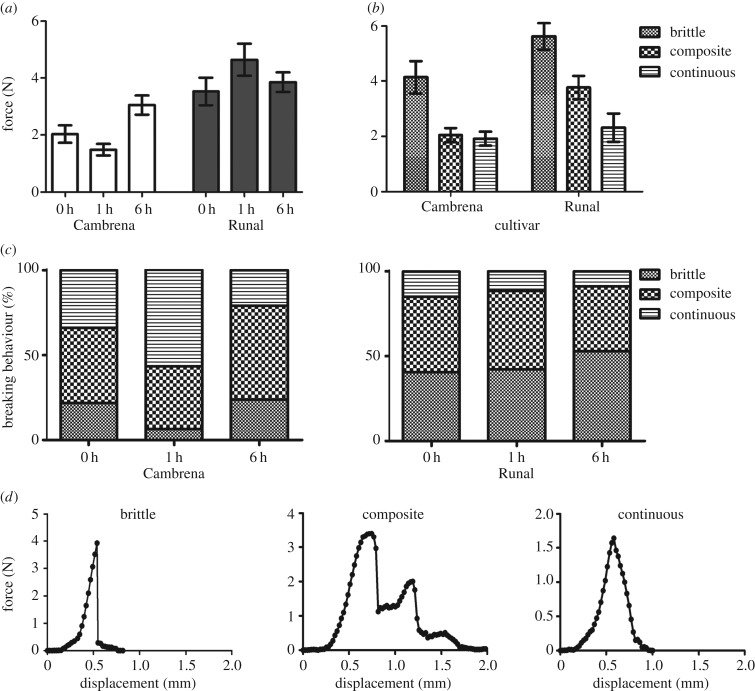
(*a*) The force required to shear the starchy endosperm of wheat grains across hard and soft cultivars at different tempering (at 16% moisture content) time points. Overall, Cambrena required significantly less force to shear than Runal (*p* < 0.001). In Cambrena, 1 h of tempering resulted in a drop in the shear force (*p* < 0.01), this was not seen in Runal. (*b*) In both Cambrena and Runal the shear forces associated with brittle failures were higher than the other categories (*p* < 0.05), except for continuous failures in Runal which were not significantly different from brittle or composite failures. The shear forces were associated with different breaking behaviours across wheat cultivars. Additionally, composite faliures required a higher force in Runal than in Cambrena (*p* < 0.001). Mean values ± s.e. shown. (*c*) Percentage of breaking behaviour in the soft wheat Cambrena and hard wheat Runal at different tempering times. The breaking behaviour pattern changes for both cultivars after 1 h tempering time. (*d*) Characteristic sample curves from earch of the three breaking behaviour categories.

Using this novel testing procedure, the force required to shear the endosperm and the corresponding breaking behaviour (figure 4) was measured using prepared cylinders of wheat samples. The grains tested were found to be unaffected by provenance but differed significantly between the two cultivars tested (*p* < 0.001), with the soft wheat, Cambrena, requiring significantly less force overall than the hard wheat, Runal (figure 4*a*). The tempering time at which shear force tests were conducted made a clear difference with the two cultivars showing a different pattern of mellowing after 1 h with Cambrena requiring less force to shear than Runal (*p* < 0.01).

The force required to shear the endosperm and the breaking behaviour observed was found to have a different relationship depending on the cultivar tested (figure 4*b*). However, the tempering time was found to have no effect and so the dataset was pooled to better assess the contrast between cultivars. In the soft wheat, Cambrena, the force recorded when a brittle failure occurred was significantly higher (*p* < 0.05) than in either composite or continuous failures which resulted in similar maximum forces. In the hard wheat, Runal, the maximum force associated with the different breaking behaviours had a different pattern with brittle failures resulting in significantly higher breaking forces when compared with continuous failures (*p* < 0.05) while composite failures required a maximum breaking force that was somewhere between the two other behaviours and not significantly different from either.

The changes in the shear force needed after 1 h tempering (figure 4*a*) coincided with a change in the breaking behaviour in Cambrena and Runal (figure 4*c*). Cambrena showed a higher percentage of continuous failures while Runal shows an increase in brittle failures at 1 h of tempering.

The three discrete breaking behaviours observed (brittle, composite and continuous) allowed for an additional means to compare hard and soft wheat grain types (figure 4*d*). If the mean forces associated with the three breaking behaviours are compared between cultivar, the brittle and continuous categories are found to be very similar. However, the force recorded when composite failures occurred was higher in Runal than Cambrena (*p* < 0.001).

**Figure 5.**
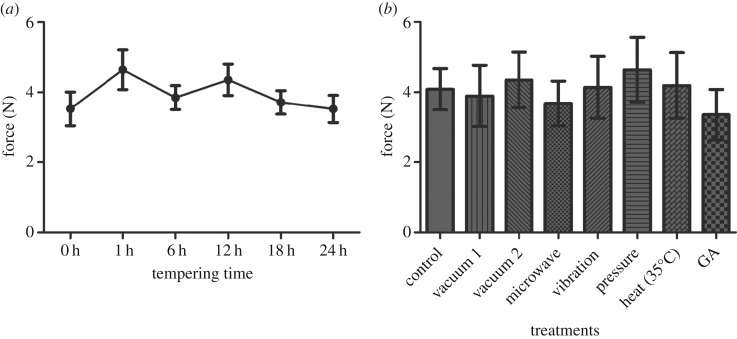
Time and treatment effects on shear forces of hard (Runal) wheat starchy endosperms during tempering. (*a*) The force required to shear the starchy endosperm of Runal grains across a 24 h tempering time course. No effect of tempering was found across the time course (*p* > 0.05). (*b*) The shear forces required to fracture the starchy endosperm in differently treated Runal 4533 grains at 6 h were not different from the control treatment (*p* > 0.05). Mean values ± s.e. shown.

A longer time course of tempering was investigated for Runal grains to reflect the typical industry standards implemented prior to milling, but despite this longer time course, there was no significant change in shear forces required to break the starchy endosperm (figure 5*a*). As no clear differences in shearing forces were observed past the 6 h time point in the tempering process further experiments focused on the first 6 h.

The hard wheat Runal of a single provenance (4533) was selected for further testing to investigate a range of pretreatments following 6 h of mellowing (figure 5*b*). Although the pretreatment procedure and measurements were successful there was found to be no significant differences between pretreatment groups and the force required to shear the starchy endosperm.

### 3.3. Energy dissipation and frequency of breaking behaviours

**Figure 6.**
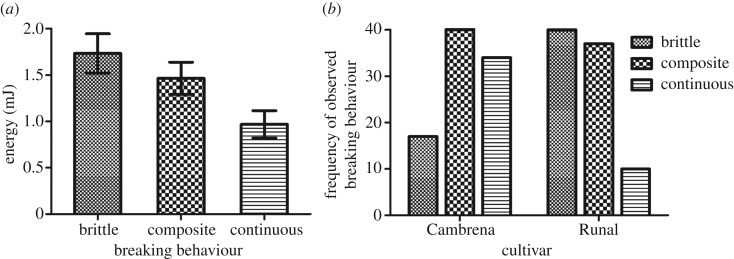
(*a*) The energy required to shear starchy endosperm was different in the three different observed breaking behaviours (cf. figure 4*c*) with brittle and composite failures requiring more energy than continuous failures (*p* < 0.05). (*b*) The frequency of the three observed breaking behaviours for the different cultivars. Runal showed more brittle failures (*p* < 0.06) and Cambrena displayed more continuous failures (*p* < 0.05). Mean values ± s.e. shown.

When the energy dissipation calculated from the experiments presented in figures 4 and 5 was analysed, it showed a close relationship with the breaking behaviour categories observed across the two grain types (figure 6*a*). There were no significant influences of cultivar, provenance or tempering time on energy dissipation. The energy involved in brittle and composite failures was found to be similar whereas continuous breaking behaviours required significantly less energy (*p* < 0.05).

Overall the frequency of different breaking behaviours was found to follow a different pattern between the two cultivars. The frequency of composite failures observed in the two cultivars was found to be extremely similar but there was, however, a trend towards more brittle failures in Runal (*p* < 0.06) and significantly more continuous failures recorded in Cambrena (*p* < 0.05). The frequencies of brittle failures in Runal and continuous failures in Cambrena match very well the frequencies of the major two vitreousness categories (table 1) with a higher number of glassy grains in Runal (*p* < 0.05), and a higher number of glassy grains in Cambrena (*p* < 0.05).

**Figure 7.**
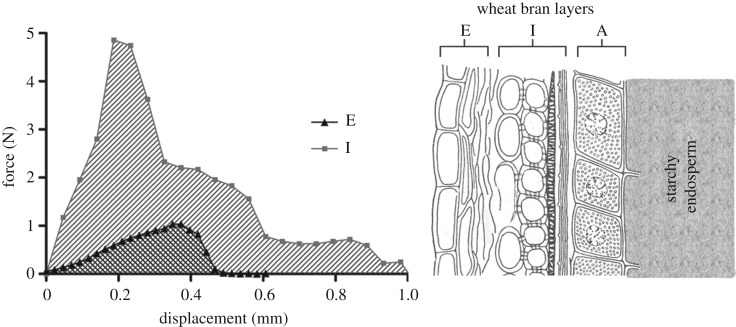
Characteristic sample force–displacement curves indicating the force required to shear the epicarp (E) and intermediate (I) layers from the aleurone (A) layer beneath, of Runal hard grained wheat. Wheat bran layers diagram after Esau [8].

A test was carried out to establish the feasibility of shearing the individual layers of bran away from the aleurone layer, which is fused to the starchy endosperm. This resulted in distinct curves (as shown in the characteristic sample curves figure 7), showing the force required to shear away the epicarp and intermediate layers. The force required to remove the Epicarp is lower than the force required to shear off the intermediate layer. This is consistent with observations during sample preparation (manual isolation of bran layers).

### 3.4. Puncture force testing of grain layer weakening

**Figure 8.**
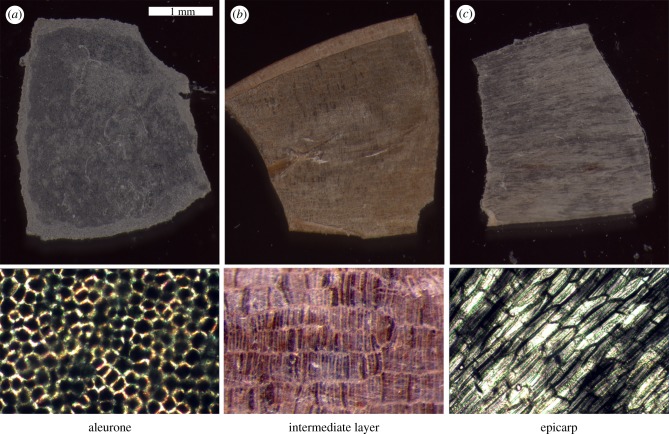
Light microscopy of manually separated bran layers of Runal wheat grains. (*a*) Aleurone layer, (*b*) intermediate layer and (*c*) epicarp.

The three separate layers (figure 8) that constitute the Runal wheat bran (figure 1*b*) were isolated and two of these layers, the aleurone and the intermediate layer were tested for mechanical resistance with the application of puncture force testing.

The force required to puncture the aleurone and intermediate layers was assessed across hormone and enzyme treatments, using methods developed specifically for this purpose (figure 3). Despite interesting effects in other treatments, the mechanical properties of the aleurone and intermediate layers were unaffected by the moisture content treatments devised to best mimic moisture contents typical of grains in the milling process (data not shown).

**Figure 9.**
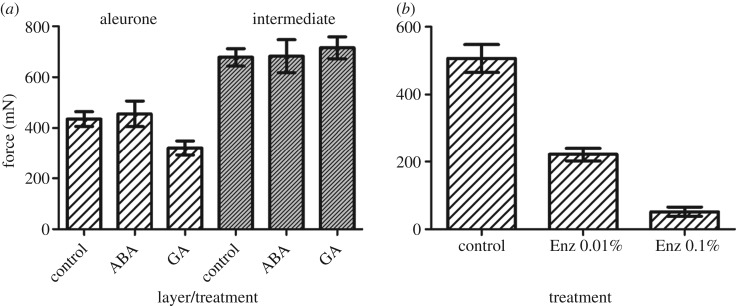
Puncture force analysis of isolated aleurone and intermediate bran layers of Runal wheat grains. (*a*) The force required to puncture bran layers across different hormone treatments; ABA, 10 µM abscisic acid; GA, 10 µM gibberellin A_4+7_. GA_4+7_ treated layers required less force to puncture than the ABA treatment (*p* < 0.05) and control treatments (*p* < 0.05). Note that the intermediate layer contains testa and inner pericarp tissues (figure 1). (*b*) The decreasing mechanical resistance of the aleurone layer to puncturing at increasing concentrations of an enzyme cocktail containing cell-wall degrading hydrolases (*p* < 0.05). Mean values ± s.e. shown.

The mechanical properties of the living aleurone tissue were influenced by hormone application (figure 9). GA application results in significantly lower tissue resistance in comparison with the ABA treatment (*p* < 0.05) and control treatments (*p* < 0.05). The difference between the two layers was significantly different in all treatments. In the intermediate layer, the measured values were not significantly different between treatments.

Gibberellins (GA) are known to induce and ABA to block the production of hydrolytic enzymes in cereal grain aleurone layers to mobilize the starchy endosperm nutrient storage during malting, preharvest sprouting (PHS) and seedling establishment [34] but their effects on the wheat grain tissue resistances has not been tested. Figure 9*a* shows that while there were no effects on the intermediate (testa plus inner pericarp) layer, treatment with GA has a strong effect on the living aleurone layer and given the strong association between GA and enzymatic activity a follow-up experiment was conducted to test the response of the aleurone layer to an enzyme cocktail containing several cell-wall hydrolases. The mechanical properties of the aleurone layer of the wheat bran were found to be modified by enzyme application (figure 9*b*). Isolated aleurone sections completely dissolved after incubation in a 1% enzyme solution. However, a 0.1% and 0.01% enzyme solution weakened the aleurone layer significantly, in a dose-dependent manner. This was indicated by the much lower force needed to penetrate the aleurone layer with enzyme solutions of 0.1% (*p* < 0.05) and 0.01% relative to untreated controls.

## 4. Discussion

The novel methodology devised for measuring the shear forces involved in different industrial processes on wheat grains enabled a number of meaningful comparisons to be drawn. The mechanical properties of wheat grains have a major impact on food science and milling performance. Milling as a ‘dry’ fractionation technology and pre-milling treatments including tempering are based on the biomechanical (texture properties) and biochemical (cell-wall composition) differences between wheat grain tissues [10]. In our study, we have developed a biomechanical assay to directly analyse the shear forces of wheat grain layers (figures 3–6) and used it to compare the tempering process of a soft with a hard grained wheat cultivar. We found clear differences in the force required to shear the two wheat cultivars, which conformed to what might be expected from a contrast between hard and soft grain types. The novel shear and puncture force methods developed in our study contribute to our understanding of the distinct tissue-scale changes during tempering of soft and hard wheat grains.

The wheat starchy endosperm is effectively a composite material in which a protein matrix holds starch granules in place [15,35]; differences in starch granule size and protein content and composition are, therefore, expected to directly influence the force required to shear this biomaterial. Hard wheat varieties tend to have a higher protein content and they have been shown to differ structurally from soft wheat varieties [2,4,15]. This is consistent with our findings for Runal and Cambrena. The hard wheat has a higher protein content and the differences in the shear force and breaking behaviour clearly indicate differences in the starchy endosperm structure. Various papers have discussed a potential relation between protein content and grain hardness, a topic recently summarized by Pasha *et al*. [3]. Starchy endosperm hardness depends on the degree of adhesion between the starch granules and the protein matrix [5]. Wang & Jeronimidis [1^5^] studied the different fracture modes and crack propagation in hard and soft wheat starchy endosperms by three-point bending tests and showed that soft wheat grains have a lower fracture toughness than hard wheats. Compression and hardness of the wheat grains have been studied extensively, and distinct mechanical properties are linked to the structure and the chemical composition of the endosperm [4,16–20]. Fracture toughness has been reported to be higher in glassy grains compared with mealy ones [2]. We found that the breaking behaviour in hard and soft wheats is different. Hard mainly glassy wheat grain endosperms tend to show brittle and composite failure types and high shear forces, while the mainly mealy endosperms tend to show continuous and composite failures. Grain hardness is conferred by the *Hardness* genes expressing two proteins, puroindoline a and b, which determine the mechanical properties of the starchy endosperm as a composite [5]. Upon milling, soft wheat produces finer flour particles which agglomerate, when compared with the coarser flour particles of hard wheat which do not agglomerate. A direct test of the shearing forces is, therefore, ideal for a comparative analysis how the starchy endosperm components separate in soft and hard wheat cultivars. Numerous papers have studied the effects of moisture content and it is one of the main factors influencing the mechanical properties of wheat grains. This has been shown by tensile tests [17,23], shear tests [36] and compression tests [21]. Nevertheless, the dry milling process does not give room for alterations in the moisture content and typically wheats are tempered to approximately 16% moisture.

Previous studies have employed different methods to assess differences between hard and soft wheat grains. The size distribution of starch granules has been used to this effect [37], with hard wheat having larger starch granules, a trait associated with larger flour yields [38] and quality [39]. Alternative methods for detecting different grain properties have used fracture mechanics and tensile testing of bran layers [23] with compression and indentation tests [16], three-point bending tests [15] and the quantification of cell-wall components [40]. When we assessed the force required to shear the starchy endosperm in wheat grains with our direct shearing assay, we found that there was a significant difference between the soft (Cambrena) and hard (Runal) grained wheat cultivars after 1 h of tempering. We found an increase in continuous failures for the soft wheat after 1 h and an increase of brittle failures in the hard wheat. Additionally, further testing on Runal showed that there were no further changes in the maximum shear force required after 1 h of tempering. This concurs with previously published data indicating that moisture content rather than tempering time has a much greater effect on subsequent flour quality [25]. The change in breaking behaviour after 1 h tempering time might implicate a structural change in the endosperm within a short time period and should be studied further including an investigation of structural endosperm composition and fracture surfaces. MRI analysis revealed that water enters the grain through the micropyle and after 2 h of imbibition water is evident in the micropylar channel, the outer pericarp and testa layers and the germ [26]. After 2 h most of the water is in the germ, but some water is distributed in the grain. Presumably, a small part of the moisture has already reached the starchy endosperm via the germ and the testa/pericarp after the first 1–2 h of imbibition. The initial moisture uptake in the starchy endosperm might contribute to a structural change and change in breaking behaviour after 1 h tempering.

With the grain that was tested, there was found to be no discernible effect of provenance on the force required to shear the wheat starchy endosperm. The five provenances tested across the two cultivars were all situated within a 200 km transect in the north-west of Switzerland and across this distance no evidence of environmental variability that may vary across such a scale was found. At much greater, continental scales the hardness of wheat has been show to vary when subjected to dramatically different climate regimes [41]. While there was no significant effect of provenance on the shear forces recorded, there was notable heterogeneity observed within the cultivars. This could be explained by the differences in grain vitreousness. At a lower moisture content of around 11% the toughness of mealy and glassy grains has been shown to differ with vitreous endosperm proving tougher than mealy endosperms [2]. To mimic industrial processes, grains conforming to these two broad categories of grain vitreousness were not separated prior to shear tests being performed, however, this property has been shown to influence the milling of wheat grains and the quality of the end product [19].

We found that various pretreatments of Runal grains make no difference to the maximum force required to shear starchy endosperm (figure 5), suggesting that as pretreatments these processes are unlikely to directly affect the physical milling process. This is in contrast to a previous study [42] looking at the effects of microwave treatments but this study used domestic appliances and estimated grinding energy using mean particle size and lacks the precision of our method. However, as the popularity of using microwave drying in agricultural products increases, it is likely that this practice may be used as a cost-effective method for rapidly and efficiently achieving required moisture contents in grains [43], a practice already implemented in maize [44] and cassava [45].

The division of breaking behaviours of the starchy endosperms of soft and hard wheat grains into three discrete categories (figure 4) was supported by the relationship between energy adsorption and the three identified breaking behaviours. The three breaking behaviours exhibited three distinct energy requirements to shear the endosperm validating the application of these three categories. The frequency of breaking behaviours observed in the two different cultivars showed that the hard and soft wheat exhibited different traits with Runal, the hard wheat, found to be more brittle and Cambrena, the soft wheat, showing more continuous failures. The differences observed between the two cultivars demonstrate that this shearing method and the identification of breaking behaviours can provide a new method for assessing the mechanical properties of wheat starchy endosperm tissues. The differences in the frequency of the breaking behaviour (more continuous in soft and more brittle in hard wheat) coincided with the vitreousness of the wheat grains. The soft wheat has a significantly higher quantity of mealy grains while the hard wheat showed a majority of glassy grains. It is known that crack propagation in the starchy endosperm of either variety occurs along the protein layer and the energy needed for fracture arises from the firmness of the adhesion between the starch granules and the protein matrix [15].

The feasibility of shearing individual layers using this technique really highlights the precision and broad applications of this method. Such a measurement would be of particular relevance to the pearling process. The pearling process [10] is an abrasive pretreatment designed to remove the outer bran layers typically leaving the aleurone layer as intact as possible. The aleurone layer is a potential source of micronutrients and technologies to enhance the exploitation of health-promoting components in whole grains are currently discussed [46]. The main benefits to pearling are when enriched grain adds value to a product, such as in spaghetti production [47] and bran-enriched breakfast cereals [48]. Being able to precisely and directly measure the shearing forces required to abrade these bran layers using our shearing force assay have the potential to increase the efficiency and yield of these methods.

The methods devised for using puncture force in characterizing the mechanical resistance of single wheat layers offers a new method and a previously unpublished measurement for wheat layers. Hitherto, this important mechanical characteristic has been approached primarily with the application of tensile testing [22,23]. Tensile tests have been carried out to determine the mechanical properties for each constitutive layer of hard and soft wheat bran [22] and the bran becomes more compliant and resilient when the moisture content increases [24]. A benefit of our puncture force method is that the sample holder applies an even force to secure samples. Additionally, using an adhesive like superglue absorbs water during hardening to keep a specimen in place would interfere with the carefully monitored water content maintained in the layers. Our puncture force method, therefore, provides an ideal platform for testing individual layers.

The ratio between the hormones ABA (inhibiting) and GA (promoting) are known to regulate endosperm weakening, an essential process during the germination of eudicot seeds as the endosperm is effectively a physical barrier to radicle protrusion [13,14]. These two hormones also regulate the germination and reserve mobilization of cereal grains in which GA serves as a signal produced by the embryo to induce the aleurone layer to express and/or secrete hydrolytic enzymes into the starchy endosperm [49–51]. In agreement with this role, the cereal aleurone is a living tissue layer of the wheat grain, but undergoes programmed cell death (PCD) during germination and seedling establishment. Coinciding with the roles of GA and ABA in germinating cereal grains, the PCD of the aleurone is promoted by GA and delayed by ABA, an observation that also has been demonstrated using isolated aleurone layers incubated in the presence of these hormones [50,51]. In agreement with these observations, we show here by puncture force measurements that GA treatment of isolated aleurone layers promotes the weakening of this living endosperm tissue, while it does not affect the dead intermediate (testa and inner pericarp) layers of wheat grains (figure 9). ABA had no effect and the GA-mediated decrease in aleurone tissue resistance was also evident upon treatment with cell-wall hydrolyzing enzymes. As for the cereal aleurone layer, which is living endosperm tissue, it is known that during the germination of eudicot seeds GA-induced cell-wall hydrolyzing enzymes are implicated in the micropylar endosperm weakening of eudicot seeds [13,14]. Also evident from these results is that endosperm weakening is delayed by ABA, and that the dead testa of these eudicot seeds does not exhibit a decreased puncture force. This is in agreement with our finding that GA treatment does not affect the resistance of the dead wheat grain intermediate layers (testa plus inner pericarp), but that the living aleurone layer weakens upon GA treatment, presumably by GA-induced cell-wall hydrolyzing enzymes.

Our new puncture and shear force testing methods may also be relevant for investigating wheat grain PHS and the late maturity alpha-amylase syndrome which cause severe reductions in grain and flour quality [34]. There is some evidence that GA and pregermination PCD are involved in the mechanisms leading to syndrome-related temporally and/or spatially misexpressed alpha-amylase. This in turn may lead to aleurone tissue weakening and altered starchy endosperm texture for which our novel puncture and shear force assays are appropriate biomechanical tools.

## 5. Conclusion

The biomechanical properties of two different wheat cultivars were investigated and new methodology developed to characterize wheat single bran layers. Meaningful comparisons were drawn for measuring the forces involved in industrial processes on wheat grain milling. Shear forces differ within soft and hard grain wheat cultivars, while the tempering process only had an influence on the first hour of the tempering process and pretreatments had no significant effect on the starchy endosperm. Endosperm weakening in the living aleurone layer depending on hormone treatments of wheat grains was demonstrated for the first time. The insights obtained in this study and the novel shear and puncture force methods developed contribute to our understanding of the distinct tissue-scale changes in the biomaterial wheat.

## Data accessibility

All raw data can be found at https://dx.doi.org/10.6084/m9.figshare.4558087.
